# What is the evidence for physical interventions for people living with advanced dementia in the community? A meta-review

**DOI:** 10.1136/spcare-2025-006058

**Published:** 2026-02-16

**Authors:** Nicola White, Alexandra Feast, Catherine Evans, Elizabeth L Sampson, Nathan Davies

**Affiliations:** 1 Marie Curie Palliative Care Research Department, Division of Psychiatry, University College London, London, England, UK; 2 Centre for Psychiatry and Mental health, Queen Mary University of London, London, England, UK; 3 Palliative Care, Policy and Rehabilitation, King’s College London, London, UK; 4 Queen Mary University of London Wolfson Institute of Population Health, London, England, UK; 5 Department of Psychological Medicine, The Royal London Hospital, London, England, UK

**Keywords:** Palliative Care

## Abstract

**Background:**

Dementia is a major global cause of death, with cases projected to rise from 57.4 million in 2019 to 152.8 million by 2050. As the disease progresses, individuals experience growing frailty, complex health needs and multiple comorbidities, requiring comprehensive support to maintain quality of care. Non-pharmacological physical interventions, targeting either the physical body or immediate environment, may help address these needs, especially in advanced dementia nearing end of life.

**Aim:**

To map, appraise and synthesise evidence on the effectiveness of physical interventions for people with dementia nearing end of life.

**Design:**

An umbrella review of systematic reviews on physical interventions, part of a broader palliative dementia care series.

**Data sources:**

MEDLINE, Epistemonikos and ASSIA were searched for systematic reviews from 1980 to May 2024 involving people with dementia, carers or staff in health and social care settings. Physical interventions were defined as non-pharmacological approaches focusing on the body or physical environment.

**Results:**

From 6052 records, 13 reviews met the inclusion criteria. Interventions grouped into two categories: (1) nutrition and hydration (including enteral feeding) and (2) physical activities (including exercise, massage and approaches to reduce care resistance). Evidence quality was generally low, with no conclusive findings on effectiveness. Most interventions addressed well-being and function, with limited evidence for carer support, holistic assessment or medical management.

**Conclusion:**

Research has primarily targeted well-being and functional outcomes, leaving key gaps in carer support and holistic management, warranting further investigation.

**Trial registration:**

PROSPERO CRD42020162887.

**WHAT IS ALREADY KNOWN ON THIS TOPIC**

- Non-pharmacological physical interventions, such as exercise and nutrition support, are commonly used to improve well-being and function in dementia care.

**WHAT THIS STUDY ADDS**

- This umbrella review suggests that existing evidence on physical interventions in advanced dementia is largely confined to nutrition, hydration and physical activity. The overall certainty of this evidence remains low, and there are notable gaps concerning carer support, holistic assessment and medical symptom management.

**HOW THIS STUDY MIGHT AFFECT RESEARCH, PRACTICE OR POLICY**

- The findings indicate a need for more robust and comprehensive research into physical interventions, particularly those that adopt holistic and carer-focused approaches in advanced dementia.
- In clinical settings, such interventions should be applied with caution and tailored to individual needs. Policy efforts may wish to consider supporting community-based care models and improving access to palliative and person-centred services for people living with dementia.

## Background

 According to the WHO there are currently 55 million people worldwide who are living with dementia and the syndrome is the seventh leading cause of death, globally.^1^

In the UK, dementia is a leading cause of death, accounting for 11% of recorded deaths,^2^ and the number of people diagnosed with dementia is set to increase by 40% (from 982 000 up to 1.4 million in 2040).^3^

Palliative care can offer holistic support for people with dementia and their family carers, addressing not only physical symptom relief but also psychological, social and spiritual support. Early referral to palliative care has been shown to help people remain in their preferred place of care and make proactive decisions about their future care, as part of their advance care planning.^4^ However, people living with dementia are less likely to be referred to specialist palliative care services partly due to the unpredictable disease trajectory and diagnostic complexities.^5^ A YouGov poll^6^ of the general population highlighted that less than half of their survey respondents were aware that dementia was a terminal condition.

Access to appropriate support in the last year of life for people with dementia remains inequitable.^7 8^ People with dementia from minority ethnic groups who live alone or have complex medical needs are more likely to experience emergency admissions to hospital in the last 12 months of life.^9^ Understanding what constitutes high quality palliative dementia care and the respective evidence is therefore essential, particularly as the new National Health Service (NHS) 10-year plan^10^ prioritises shifting care from the hospital to the community.

This review forms part of a larger programme of work which aims to identify interventions to improve end-of-life care for people with advanced dementia living in the community. It specifically focuses on interventions related to physical interventions. These are non-pharmacological interventions that involve direct physical care aimed at relieving symptoms and promoting comfort. They focus on the patient’s body and physical environmental needs, addressing issues such as pain, breathlessness, skin integrity, nutrition and hydration. These may include manual therapy (eg, a massage), nutritional support, adaptive equipment to the immediate environment (eg, mobility aids or coloured plates). These interventions can help to improve physical well-being (eg, balance, strength), cognition and independence in activities of daily living.^11^

By understanding what the evidence is for interventions to address physical health needs, clear recommendations for research, clinical practice and guidelines can be developed. Given the substantial body of synthesised data that already exists on this topic, an umbrella review is the most appropriate approach to capture and assess the breadth, consistency and quality of the available evidence and to highlight any existing gaps.^12^

The aim was to map, appraise and synthesise the evidence on effectiveness of physical interventions for people living with dementia nearing the end of life.

Objectives:

1. To describe the physical interventions used to support people with dementia nearing the end of life.
2. To examine the stated outcomes and assess the effectiveness of these interventions addressing physical health or symptoms.
3. To identify gaps in the evidence related to interventions to support physical needs in end-of-life dementia care.

## Methods

### Design

This umbrella review was guided by the Cochrane Handbook^13^ and reported using the Preferred Reporting Items for Systematic Reviews and Meta-Analysis (PRISMA) statement.^14^ This review formed part of the evidence synthesis work stream of the Empowering Better End of Life Dementia Care programme of research (EMBED-Care).^15^ There is a published protocol for this review on PROSPERO (CRD42020162887), which describes the overall aim of a series of reviews looking more broadly at palliative care interventions in dementia care; however, due to the complexity of the literature, separate reviews were completed focussing on different types of interventions for palliative dementia care, for example, psychosocial^16^ and carer interventions. This review focuses on physical interventions for people living with advanced dementia.

### Eligibility criteria

The criteria for the overall aim of the series of reviews were the following:

#### Inclusion

- ≥50% of studies with participants with dementia/cognitive disorders, considered in the last 1 or 2 years of life, or those in the moderate to advanced stages of dementia as rated by validated tools (Functional Assessment Staging Tool (FAST) stage 5 or above; Clinical Dementia Rating 2 or above; or Mini Mental State Examination 0–10 severe; 11–20 moderate).
- Carers of people with dementia, including family carers (including friends) and paid carers (eg, home care staff).
- Studies in health and social care including hospital (eg, inpatient, emergency department, outpatient), primary care, community services (eg, mental health, home nursing), hospice (eg, inpatient hospice, hospice home-care), care homes (eg, nursing home, residential care home, long-term care), social care in personal dwelling (eg, at home).
- Palliative care interventions using the WHO definition of palliative care.^17^
- Any comparator (including usual care, active control or no comparator).
- Systematic reviews which searched more than one database, included an aim of effectiveness and included more than one study.

Systematic reviews of qualitative and quantitative studies were included. The purpose of this was to explore how people experience the intervention in addition to understanding whether an intervention works (or not).

For this review, studies were then further screened to determine whether they were a physical intervention. A physical intervention was defined as: any non-pharmacological intervention that provided direct physical care, either to the physical body or on the immediate physical environment. Reviews that did not have this focus were not included.

#### Exclusion

Reviews describing only case studies or series, or descriptive studies about patient/carer needs or experiences of dementia and scoping reviews were ineligible. Pharmacological and post-death interventions are outside the scope of the review. There were no language restrictions.

### Information sources

Three electronic databases (MEDLINE, health and social care data; Epistemonikos, systematic reviews; ASSIA, social care systematic reviews) were searched for systematic reviews published between January 1980 and May 2024. This starting date aligns with key evidence on the need for hospice care for people with advanced dementia.^18^

### Search Strategy

Search terms were developed and refined in collaboration with an expert systematic reviewer (BC) and previous literature. The search domains included: dementia, palliative care, systematic reviews. See online supplemental material 1 for the search terms for each database. Forward and backward citation searches were conducted, as well as contact with key authors in the field to identify additional work.

### Selection

Two researchers (BC, TA, YBM or SM) screened the search results against the eligibility criteria. Any discrepancies were resolved by a meeting with the two reviewers in the first instance. If unresolved, a third reviewer (CE, ELS or ND) was consulted. EndNote X8 was used for the management of the search results. Prior to screening, 170 duplicates were identified and removed automatically via EndNote X8. Manual checking for duplicates occurred during the screening process.

An Excel sheet was piloted for data extraction for the overall review. For this specific review on physical healthcare interventions, the researcher (NW) extracted data from each included review adhering to the same excel sheet.

### Quality appraisal

Included studies were rated for their quality using the AMSTAR 2^19^ (quantitative reviews) or ENTREQ^20^ (qualitative reviews). The following criteria were adopted:

**High -** Zero or one non-critical weakness: The systematic review provides an accurate and comprehensive summary of the results of the available studies that address the question of interest

**Moderate -** More than one non-critical weakness: The systematic review has more than one weakness, but no critical flaws. It may provide an accurate summary of the results of the available studies that were included in the review.

**Low -** One critical flaw with or without non-critical weaknesses: The review has a critical flaw and may not provide an accurate and comprehensive summary of the available studies that address the question of interest.

**Critically low -** More than one critical flaw with or without non-critical weaknesses: The review has more than one critical flaw and should not be relied on to provide an accurate and comprehensive summary of the available studies. 

### Data Synthesis

For each review, the following data were extracted:

1. Details about the publication (first author, year, location).
2. Details of the review (focus, primary aim, design).
3. Details about the search (study designs included, databases searched, date range, number of studies identified).
4. Details about the participants (setting, participants).
5. Details about the intervention.
6. Details about the outcome and analysis.

In reviews where a meta-analysis was conducted, data were extracted regarding the sample size, effect size and heterogeneity. These data are tabulated in this review. Primary studies cited in the reviews were cross-checked for duplication across the reviews and reported, using the Graphical Representation of Overlap for OVErviews tool.^21^ This tool calculates the corrected covered area (CCA) score through the number of primary studies, the number of reviews and the number of instances where studies are reported in more than one review. This results in a percentage score, where <5% indicates a slight overlap, 5% to <10% is moderate, 10% to <15% is high, ≥15% is very high overlap. Any review that was identified as ‘very high’ was compared during the analysis.

The Grading of Recommendations Assessment, Development and Evaluation (GRADE) assessment was used for outcomes identified in a meta-analysis. The criteria for each domain were determined from previous literature and are reported in online supplemental material 2.

Where no meta-analysis was conducted, the main results are described.

The interventions described in the reviews were mapped on to a modified framework of key components of good palliative and good dementia care domains, a method previously adopted for psychosocial interventions in palliative dementia care.^16^ As the focus on this review was on physical interventions, the concepts from the initial paper (13 were generated) were reduced to 9, excluding (1) continuity of care and care coordination, (2) managing care transitions, (3) diagnosis and prognosis, (4) pharmacological treatments. As done previously, the evidence included from this review was mapped on these nine domains. This process identified current evidence and subsequent gaps regarding physical interventions. This process was validated by an independent reviewer (AF).

## Results

**Figure 1:**
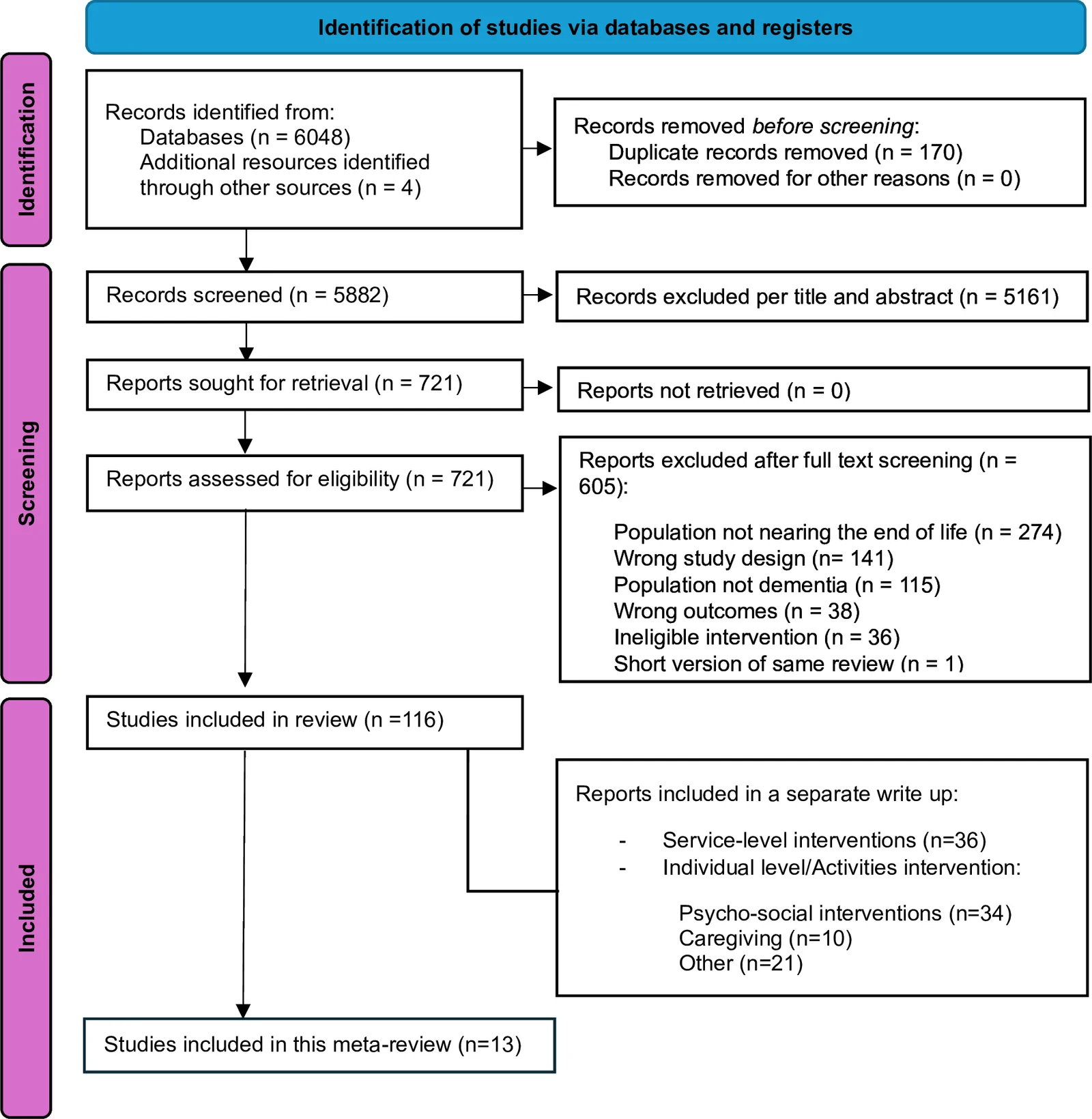
Adapted from PRISMA 2020 flow diagram. PRISMA, Preferred Reporting Items for Systematic Reviews and Meta-Analysis.

In total, 6052 records were identified from the databases and additional resources. After screening and removing duplicates, 721 records were assessed for eligibility. After full text screening, 116 records were included in the review: 13 of which focused on intervention aimed to address healthcare needs (see figure 1).

Of the 13 included reviews, four were Cochrane systematic reviews,^22–25^ 6 systematic reviews,^26–31^ 2 qualitative reviews^32 33^ and 1 best-evidence review.^34^ Three reviews included meta-analysis.^23 24 31^ Five reviews were conducted in the UK,^22 25 28 32 33^ two in the USA,^2 29 30^ two in Germany,^24 31^ one in Australia,^27^ Canada,^23^ Ireland^26^ and Japan.^34^ Table 1 describes the aims and population of each included review.

**Table 1:** Overview of the included reviews

Author	Year	Location	Focus	Primary aim of review	Participants	Setting
Sampson *et al*^25^	2009	UK	Enteral feeding	To evaluate the outcome of enteral tube nutrition for older people with advanced dementia who develop problems with eating and swallowing or who have poor nutritional intake.	Advanced dementia	Any
Davies *et al*^22^	2021	UK	Enteral feeding	To assess the effectiveness and safety of enteral tube feeding for people with severe dementia who develop problems with eating and swallowing or who have reduced food and fluid intake.	Advanced dementia	Healthcare Long-term care Own home
Bunn *et al*^28^	2015	UK	Hydration	To assess the effectiveness of interventions and environmental factors to increase fluid intake or hydration status in older people living in long-term care	≥65 years	Residential Long-term nursing care Specialist dementia units
Wilson and Dewing^33^	2020	UK	Hydration	To determine the evidence-based interventions used to prevent and manage dehydration in older people with dementia.	Older people with dementia	Any
Liu *et al*^29^	2014	USA	Nutrition	To evaluate the effects of interventions on mealtime difficulties in older adults with dementia.	≥65 years Dementia	Any
Liu *et al*^30^	2015	USA	Nutrition	To summarise available interventions and evaluate their effectiveness on eating performance among older adults with dementia in long-term care settings.	≥65 years Dementia	Long-term care setting
Herke *et al*^24^	2018	Germany	Nutrition and hydration	To assess the effects of environmental or behavioural modifications on food and fluid intake and nutritional status in people with dementia.	Alzheimer’s Vascular Lewy body Parkinson’s Frontotemporal	Any
Barrado-Martin *et al*^32^	2021	UK	Nutrition and hydration	To synthesise the qualitative evidence of the views and experiences of people living with dementia, family carers and practitioners on practice related to nutrition and hydration of people living with dementia near the end of life.	End-of-life dementia	Any
Brett *et al*^27^	2016	Australia	Physical exercise	To evaluate the effects of physical exercise interventions on individuals living with a dementia in nursing homes.	Dementia	Long-term care setting
Forbes *et al*^23^	2008	Canada	Physical exercise	Do physical activity programmes manage or improve cognition, function (eg, activities of daily living), behaviour, depression and mortality compared with usual care in older persons with dementia?	≥65 years Dementia	Long-term care setting
Barrett *et al*^26^	2021	Ireland	Physical exercise	To examine the effectiveness of function-oriented physical activity programmes on the physical, psychological, cognitive and adverse outcomes of older adults living in nursing homes	≥60 years	Nursing home setting
Margenfeld *et al*^31^	2019	Germany	Massage	To pool the evidence for the efficacy of manual massage for persons living with dementia	Dementia	Any
Konno *et al*^34^	2014	Japan	Resistance-to-care	To review the evidence of non-pharmacological interventions for resistance-to-care behaviours of nursing home residents with dementia in a personal-care context.	≥55 years Dementia	Residential care setting

* ENTREQ quality rating measure. All others AMSTAR.

Table 2 describes the characteristics of the systematic reviews. Quality appraisal of the reviews indicated one review was rated as high quality,^24^ seven were moderate^22 25 28 29 31 32 34^ and five were low quality.^23 26 27 30 33^ Specific to the rating domains, the source of funding and the duplication processes were considered the most at risk. See online supplemental material 3 for the breakdown by domain.

**Table 2:** Overview of the systematic review characteristics

Author	Type of review	Study designs included	Number of databases searched	Date range of search	Studies	Quality
Sampson *et al*^25^	Cochrane review	RCT Controlled before–after ITT Controlled observational	7	December 2005 to April 2008	7	Moderate
Davies *et al*^22^	Cochrane review	RCTs Controlled non-randomised	7	December 2019 to April 2021	14	Moderate
Bunn *et al*^28^	Systematic	Interventional Observational	13	Inception to September 2013	23	Moderate
Wilson and Dewing^33^	Literature review	Any	3	2001 to December 2018	12	Low*
Liu *et al*^29^	Systematic	Interventional Experimental Quasi-experimental	5	January 2004 to September 2012	22	Moderate
Liu *et al*^30^	Systematic	Interventional Experimental Quasi-experimental	5	January 1980 to June 2014	11	Low
Herke *et al*^24^	Cochrane review	RCTs Cluster RCTs Cross-over trials	7	Inception to January 2018	9	High
Barrado-Martin *et al*^32^	Qualitative systematic	Qualitative	4	January 2000 to February 2020	20	Moderate*
Brett *et al*^27^	Systematic	RCTs Cluster RCTs	12	Not described	12	Low
Forbes *et al*^23^	Cochrane review	RCTs Cross-over trials	7	Inception to September 2007	4	Low
Barrett *et al*^26^	Systematic	RCTs Cluster RCTs non-randomised before-and-after	8	Inception to June 2018	23	Low
Margenfeld *et al*^31^	Systematic	RCTs	9	Inception to August 2017	11	Moderate
Konno *et al*^34^	Best evidence review	RCT Quasi-experimental studies	9	1990–2012	19	Moderate

* ENTREQ quality rating measure. All others AMSTAR.

ITT, Intention-To-Treat; RCT, Randomised Controlled Trial.

Table 2 describes the quality appraisal of the included reviews and their conclusions. In total, 187 citations are reported across the reviews, of which 120 (64%) were categorised as ‘high risk’. Three of the included reviews reported data from a meta-analysis.^23 24 31^

There were 20 primary studies reported in more than one review. The CCA score was 1.10%, indicating slight overlap overall. There were two instances where the CCA indicated a very high overlap on individual reviews. On inspection, two reviews with a CCA of 16.7% had the same review question, with the most recent review^22^ being an update of the first.^25^ Two other reviews were identified as having very high overlap.^26 27^ Both reviews are looking at physical exercise programmes for older adults living in nursing homes, with a 5-year publishing gap. These overlaps were considered in the interpretation of the data and in the discussion. See online supplemental material 4 for the full CAA analysis.

The interventions detailed in the 13 reviews were categorised into two main areas: (1) nutrition and hydration and (2) physical activities.

### Nutrition and hydration

Most physical interventions concerned nutrition and hydration. Eight reviews (reporting data from 118 studies) focused on interventions supporting hydration and/or nutrition.^22 24 25 28–30 32 33^ Two focused specifically on enteral feeding,^22 25^ two focused only on hydration,^28 33^ two focused only on nutrition^2 29 30^ and two studies focused on nutrition and hydration.^24 32^

#### Nutrition and Hydration

The two reviews address different components. Herke *et al*^24^ examined the effects of environmental and behavioural modifications on food and fluid intake; Barrado-Martin *et al*^32^ explored views and experiences of nutrition and hydration at the end of life.

Herke *et al*^24^ synthesised findings from nine studies on interventions aimed at improving food and fluid intake through environmental and behavioural strategies. These included:

1. Additional food items between meals compared with no intervention.
2. Educational and nutritional programme compared with no intervention.
3. Space retrieval (a memory training technique) combined with errorless learning training compared with spaced retrieval only.
4. Spaced retrieval compared with no intervention.
5. Montessori-based activity (structured, meaningful, hands-on task) compared with no intervention.
6. Feeding skills training for nurses compared with no intervention.
7. Verbal and physical encouragement compared with only verbal encouragement.

Using the GRADE system,^35^ the authors reported limited or little confidence in most study results. No specific environmental or behavioural modifications were identified as effective for improving food and fluid intake. All included studies were judged to have domains at high risk of bias. Evidence from a meta-analysis was presented (see table 3). Across the included studies, 37 different outcomes were extracted from seven interventions (see table 4). Although the overall quality of the review was high, the meta-analyses largely relied on data from a single study for all but one outcome—body mass index (BMI) at 12 months—which was not statistically significant.

**Table 3:** Evidence rating

Intervention	Author	Studies	Meta-analysis	Quality rating of the review	Studies at high risk of bias	Review conclusion
**Nutrition and hydration**	Herke *et al*^24^	9	Yes	High	9/9 (100%)*	See meta-analysis
	Barrado-Martin *et al*^32^	20	No	Moderate	20/20 (100%)†	Four themes discussed from the data
**Enteral feeding**	Sampson *et al*^25^	7	No	Moderate	7/7 (100%)‡	Insufficient evidence
	Davies *et al* ^22^	14	No	Moderate	11/14 (79%)§	Insufficient evidence
**Nutrition**	Liu *et al* ^29^	22	No	Moderate	3/22 (14%)¶	Moderate evidence for ‘Nutritional supplements’ and ‘Training/education programmes’
	Liu *et al*^30^	11	No	Low	3/11 (27%)¶	Moderate evidence for using training programmes and mealtime assistance to optimise eating performance
**Hydration**	Bunn *et al*^28^	23	No	Moderate	23/23 (100%)*	Limited evidence
	Wilson and Dewing^33^	12	No	Low	Not completed	Five themes discussed from the data
**Physical exercise**	Brett *et al*^27^	12	No	Low	3/12 (25%)**	Emerging evidence that physical exercise has a positive effect on health and well-being
	Forbes *et al*^23^	4	Yes	Low	3/4 (75%)††	See meta-analysis
	Barrett *et al*^26^	23	No	Low	17/23 (74%)	Walking interventions and functional exercise programmes may be effective for improving physical outcomes in nursing home residents
**Massage**	Margenfeld *et al*^31^	11	Yes	Moderate	2/11 (18%)	See meta-analysis
**Resistance-to-care**	Konno *et al*^34^	19	No	Moderate	19/19 (100%)*	Low quality of evidence

* No overall score, but all included domains with high risk of bias.

† No study reported all domains of quality assessment.

‡ Authors report ‘All studies have a high risk of bias by the nature of their research design’.

§ Study rated as either critical or serious.

¶ Rated as weak.

** Scored less than 5 (authors cut-off value for high risk).

†† Rated high or moderate risk.

**Table 4:** Meta-analysis data with outcomes

Review	Intervention	Outcome	Studies	Sample size		Meta-analysis data		
				Intervention	Control	Effect	Overall effect (Z)	P value
Margenfeld *et al*^31^ Quality rated: moderate	Manual massage	CMAI	6	188	207	−0.56 (−0.95 to –0.17)	2.81	0.005
		NPI	3	34	37	−16.67 (−35.39 to 2.05)	1.75	0.08
		Cornell Scale of Depression.	3	89	104	−6.08 (−8.74 to –3.43)	4.49	<0.00001
Forbes *et al*^23^ Quality rated: low	Physical activity	Function at 7 weeks-6 months (CADS and ADL).	2	62	66	−0.08 (−0.68 to 0.52)	0.26	0.79
		Function from baseline to 12 months (ADL).	1	56	54	0.3 (−0.13 to 0.73)	1.37	0.17
		Behavioural disturbances at 6 months (NPI).	1	60	57	−1.0 (−3.96 to 1.96)	0.66	0.51
		Behavioural disturbances at 12 months (NPI).	1	56	54	−0.6 (−4.22 to 3.02)	0.32	0.75
		Depression at 6 months, (MADRS).	1	60	57	−1.8 (−4.14 to 0.54)	1.51	0.13
		Depression at 12 months, (MADRS).	1	56	54	−1.4 (−4.24 to 1.44)	0.97	0.33
Herke *et al*^24^ Quality rated: high	Additional food	Calories consumed per meal (kcal, 6 weeks).	1	24	18	−50.00 (−286.41 to 186.41)	0.41	0.68
		Calories consumed between meals (kcal, 6 weeks).	1	24	18	231.00 (123.98 to 338.02)	4.23	0.000023
		Calories consumed in total (kcal, 6 weeks).	1	24	18	181.00 (−103.08 to 465.08)	1.25	0.21
		Body weight (kg, 6 weeks).	1	24	18	−0.22 (−1.25 to 0.81)	0.42	0.68
	Education and nutrition programme	Total protein intake (g/kg of body weight, 12 months).	1	40	38	0.11 (−0.01 to 0.23)	1.77	0.078
		Mini Nutritional Assessment (MNA, months).	1	291	365	−0.1 (−0.67 to 0.47)	0.34	0.73
		Body mass index (BMI, 6 months).	1	25	27	−1.79 (−2.3 to –1.28)	6.92	<0.00001
		BMI (12 months).	2	331	403	−0.26 (−0.70 to 0.19)	1.14	0.25
		Body weight (kg, 6 months).	1	25	27	−8.11 (12.56 to –3.66)	3.58	0.00035
		Body weight (kg, 12 months).	1	291	365	−1.6 (−3.47 to 0.27)	1.68	0.094
		Arm muscle circumference (cm, 6 months).	1	25	27	−1.3 (−1.78 to –0.82)	5.26	<0.00001
		Arm circumference (cm, 6 months).	1	25	27	0.24 (0.12 to 0.36)	3.78	0.00016
		Triceps skinfold change scores (cm, 6 months).	1	25	27	−0.46 (−2.67 to 1.75)	0.41	0.68
		Eating Behaviour Scale (12 months).	1	291	365	−1.5 (−2.11 to –0.89)	4.82	<0.00001
		Mini Mental State Examination (MMSE, 12 months).	1	291	365	−1.5 (−2.52 to –0.48)	2.87	0.0041
		Activities of daily living scale (ADL, 12 months).	1	291	365	−0.65 (−0.93 to –0.37)	4.49	<0.00001
		Instrumental activities of daily living (12 months).	1	291	365	−0.45 (−0.80 to –0.10)	2.49	0.013
		Clinical Dementia Rating global score (12 months).	1	291	365	0.13 (0.02 to 0.24)	2.25	0.025
		Neuropsychiatric Inventory Questionnaire (NPI-Q, 12 months).	1	291	365	0.7 (−0.12 to 1.52)	1.67	0.094
		Health-related quality of life (change scores).	1	40	38	0.02 (−0.02 to 0.06)	1.06	0.29
		Falls per year (falls/person, 12 months).	1	40	38	−0.84 (−1.31 to –0.37)	3.51	0.00045
	Spaced retrieval with errorless learning	Amount eaten (percentage, 8 weeks).	1	31	29	−5.6 (−11.70 to 0.50)	1.8	0.072
		MMSE (8 weeks).	1	28	25	2.5 (−0.46 to 5.46)	1.65	0.098
	Spaced retrieval training programme	Amount eaten (percentage, 3 months).	1	31	23	2.67 (−5.22 to 10.56)	0.66	0.51
		Body weight (kg, 6 months).	1	19	14	3.68 (1.88 to 5.48)	4.01	0.0006
		BMI (8 weeks).	1	19	14	1.73 (−0.63 to 4.09)	1.44	0.15
		Body weight (kg, 8 weeks).	1	19	14	3.35 (−2.72 to 9.42)	1.08	0.28
		Edinburgh Feeding Evaluation in Dementia scale (EdFED, 8 weeks).	1	31	23	−1.67 (−2.34 to 1.00)	4.91	<0.00001
	Montessori-based activities training programme	Amount eaten (percentage, 3 months).	1	28	23	−9.96 (−17.86 to –1.52)	2.33	0.02
		MNA (8 weeks).	1	17	14	−2.31 (−4.62 to 0.00)	1.96	0.05
		BMI (8 weeks).	1	17	14	−1.94 (−3.95 to 0.07)	1.89	0.059
		Body weight (kg, 8 weeks).	1	17	14	−3.93 (−9.62 to 1.76)	1.35	0.18
		EdFED (8 weeks).	1	28	23	−1.5 (−2.16 to –0.84)	4.45	<0.00001
	Feeding skills training programme for nurses	Amount eaten (percentage, 3 months).	1	12	8	−9 (−27.86 to 9.86)	0.94	0.35
		EdFED (3 months).	1	12	8	2.3 (0.26 to 4.34)	2.21	0.027
	Verbal encouragement and physical encouragement by touch	Calories consumed per meal (kcal, 3 weeks).	1	21	21	200.00 (119.81 to 280.19)	4.89	<0.00001
		Protein consumed per meal (grams, 3 weeks).	1	21	21	15 (7.74 to 22.26)	4.05	0.000052

CADS, Changes in Advanced Dementia Scale; CMAI, Cohen-Mansfield Agitation Inventory; MADRS, Montgomery-Åsberg Depression Rating Scale.

Barrado-Martin *et al*^32^ aimed to synthesise the qualitative evidence on the views and experiences of multiple perspectives on practices related to nutrition and hydration at the end of life. They identified 20 studies and described the following four themes:

1. Challenges of supporting nutrition and hydration: This theme discussed difficulties with communication, a lack of preparation, strain on carers and a lack of information or understanding.
2. Balancing the views of all parties involved: This theme discussed disagreements and conflicts, ethical concerns and issues with prognosis.
3. National context and sociocultural influences: This theme discussed national factors (eg, resources, regulations) and cultural values internationally.
4. Strategies to support nutrition and hydration near the end of life in dementia: This theme discussed strategies identified to support nutrition and hydration. This included getting to know the person, enriching food, texture modified diets, assisted techniques, environmental and contextual modifications.

The authors concluded that supporting nutrition and hydration at the end of life is inherently complex. This complexity arises not only from clinical uncertainty and practical challenges but also from managing differing perspectives and navigating the influence of national policies and structural factors such as funding.

#### Enteral feeding

Davies *et al*^22^ in an update to the Sampson *et al*^25^ Cochrane review conducted three comparisons: comparing percutaneous endoscopic gastrostomy (PEG) to no enteral tube feeding; nasogastric tube feeding versus no enteral tube feeding, and mixed (nasogastric, PEG or unspecified) versus no enteral feeding. The authors report that 79% of the studies were either critical or serious for risk of bias (see table 2). Using the same outcomes as the Sampson review, Davies reported that there was no conclusive evidence that enteral tube feeding provided any benefit for people with severe dementia. They reported that only one study reported pain as an outcome which found no difference between groups. However, they did find some evidence of a clinically significant risk of pressure ulcers from enteral tube feeding.

#### Nutrition

Two reviews, based in the USA by the same lead author, focused on the impact of interventions on nutrition.^29 30^ The aim of the 2014 review was to look at interventions for mealtime difficulties for people with dementia in general. The 2015 review focused specifically on long-term care settings. Liu 2014 identified 22 studies; Liu 2015 identified 11 studies (4 of these studies were included in both reviews). Liu 2014 identified five domains for interventions: nutritional supplements, training/educational programmes, environmental/routine modifications, feeding assistance, mixed interventions.

Liu *et al*^29^ analysed the data by six main outcomes, using the GRADE scoring system.^35^ The main outcomes were body weight, BMI, food intake, eating time, feeding difficulty and agitation. The authors concluded for the respective outcome that there was:

1. Body weight and BMI: Moderate evidence to support the use of nutritional supplements to increase body weight and BMI.
2. Food intake: Moderate evidence for nutritional supplements, low evidence for environment/routine, insufficient evidence for training/educational programmes and feeding assistance.
3. Feeding difficulty and eating time: Moderate evidence for training/education programmes for staff to increase eating time and decrease feeding difficulties.
4. Agitation: Insufficient evidence that relaxing or soothing music during mealtimes decreases agitated behaviours.

They reported that three studies (14%) were rated as ‘weak’ for their risk of bias (see table 2).

Lin *et al* review^30^ qualitatively aggregated the data to address four key intervention styles that couple optimising eating performance: training programmes for staff, mealtime assistance, environmental modifications and multicomponent interventions. They rated three studies (27%) as ‘weak’ for their risk of bias assessment (see table 2). The authors conclude that there is preliminary evidence to support the use of training programmes and mealtime assistance to optimise eating performance for people with dementia in a long-term care setting.

#### Hydration

Two reviews focused on hydration only,^28 33^ including 23 and 12 studies, respectively, with 5 studies appearing in both.

Bunn *et al*^28^ aimed to assess the efficacy of interventions and environmental factors on increasing fluid intake or reducing dehydration risk in older people living in long-term care facilities. The results are reported across eight themes: drinking vessel characteristics, drink characteristics, physical and social setting for drink, institutional factors (minimum data set), institutional factors (staffing; ownership and type of facility; size and location), care aimed at increasing fluid intake. The authors noted that all studies were at high risk of bias (see table 2). While they found some trends that interventions can increase fluid intake, the low quality of the evidence (high risk of bias) means they are unable to demonstrate the effectiveness of the interventions.

Wilson and Dewing^33^ reviewed strategies to prevent dehydration in people living with dementia. They presented five key themes that were strategies considered to improve hydration: physical and social environment, staff communication strategies, access to drinks, drinking vessels and individual preferences. No risk of bias assessment was reported with this review.

### Physical activities

Five reviews focused on interventions related to exercise.^23 26 27 31 34^ Three studies focused on physical exercise,^23 26 27^ one study focused on massage^31^ and one on interventions to minimise resistance to care behaviours.^34^

#### Physical Exercise interventions

Brett *et al*^27^ included 12 studies in their review, 3 of which were considered high risk of bias (see table 2). Their aim was to evaluate the effects of physical exercise on health and well-being of people living with dementia in nursing homes. They categorise the interventions as: multimodal (six studies), walking only (five studies), music and movement (two studies), hand exercises (one study). The authors reported the outcomes used across the studies, categorising them broadly as: cognitive function (10 studies), mood and depression (8 studies), functional ability (5 studies), mobility (4 studies) and unmet needs (4 studies). The authors did not conduct a meta-analysis of the data, but report that there is emerging evidence that physical exercise can have a positive effect on health, particularly the multimodal interventions. They state that 9 of the 12 studies showed a significant improvement in the intervention and/or deterioration in the control group.

Forbes *et al*^23^ primary aim was to assess if physical activity programmes could manage or improve cognition, function, behaviour, depression and mortality compared with usual care in people living with dementia living in the community or long-term care. They identified four studies, two of which were included in a meta-analysis. The meta-analysis showed no significant findings on function, which was the only outcome included in the meta-analysis (see table 4). The authors concluded that there was insufficient evidence of benefit in managing or improving cognition, function, behaviour, depression and mortality in people with dementia.

Barrett *et al*^26^ examined the effectiveness of function-orientated physical activity programmes embedded within the care of older adults living in nursing homes. The authors included 23 studies, of which all were considered to have high risk of bias (see table 2). Interventions (functional exercise programmes, activity of daily living practice, walking programmes) were reported by the following outcomes: physical, psychological, cognitive and adverse events. There was no meta-analysis conducted due to heterogeneity (content, duration, comparators and populations), so a narrative synthesis was conducted. The authors concluded that: (1) There is some evidence that functional exercise programmes can improve physical and cognitive outcomes. (2) No intervention improved psychological outcomes. (3) Adverse events did not increase following any intervention.

#### Massage

Margenfeld *et al*^31^ in their meta-analysis aimed to assess the effectiveness of manual massage for people living with dementia. They identified 11 studies. Outcomes were validated measures for agitation and cognition. The authors pooled the data to conduct a meta-analysis on n=9 studies (see table 4), whereby the evidence suggests that manual massage improved depression and agitation. However, there was significant heterogeneity with agitation.

The authors conclude that a manual massage can improve behavioural and psychological symptoms of dementia as shown by the Cohen Mansfield Agitation score and Cornell Scale, but not the Neuropsychiatric Inventory group.

#### Minimising resistance to care behaviours

Konno *et al*^34^ conducted a review to assess the evidence for non-pharmacological interventions to minimise resistance to care behaviours, during personal care in a nursing home. The authors describe ‘resistance-to-care’ as ‘any patient behaviour which prevents or interferes with the nurse performing or assisting with activities of daily living for the patient, including bathing, toileting and grooming’. The review included 19 studies, all of which were rated as high risk of bias (see table 2). They could not conduct a meta-analysis on the data due to clinical heterogeneity. Outcome measures were focused on measuring disruptive behaviour, problem behaviour, agitation, aggression and resistance-to-care.

The authors present the findings in two domains: (1) environmental interventions, in which music was commonly played to reduce resistance to care behaviours, and (2) educational programmes for caregivers which were described in two subdomains: person-centred care for bathing or an educational programme with an ability-focused approach for daily basic care. The authors concluded that while most studies across the two domains showed a trend for improvement in behaviours through non-pharmacological interventions, the quality of the evidence was low.

### Certainty of the evidence

Online supplemental material 2 gives the GRADE assessment and criteria for rating. There were 12 outcomes included in the meta-analyses regarding the impact of physical interventions. All 12 outcomes were graded as very low certainty of the evidence. This was primarily due to the high risk of bias and the inconsistency in the reporting of the analyses.

### Mapping the interventions on to the domains of palliative and dementia care

**Figure 2:**
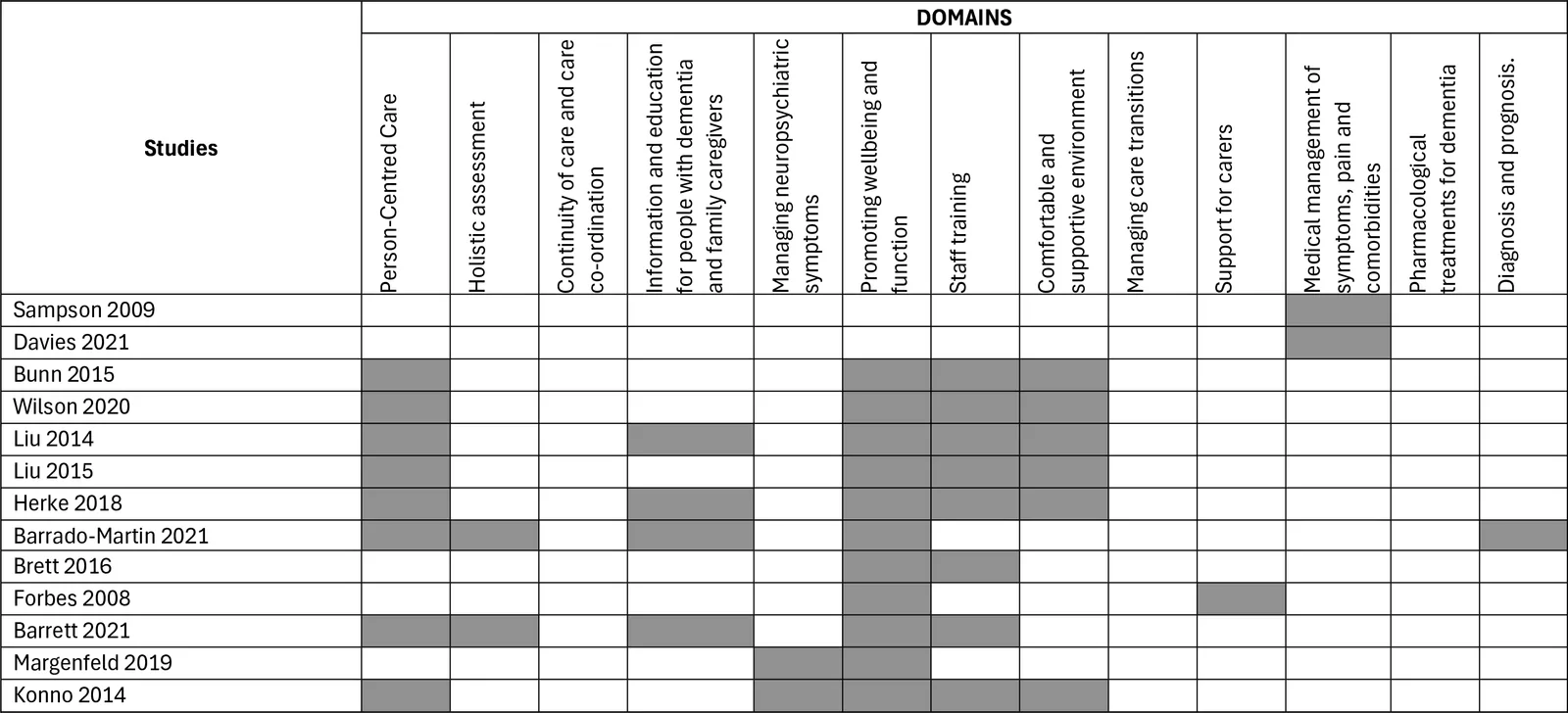
Mapping the reviews on to the domains.

Figure 2 maps the interventions from each review on to the framework of key components related to good palliative and dementia-related care. No domain was covered by all reviews. The domain of ‘Promoting wellbeing and function’ was covered the most, by 11 of the 13 reviews. Support for carers (n=2), holistic assessment (such as advance care plans) (n=2) and the medical management of symptoms (n=2) were the least represented domains, suggesting gaps in the evidence for these areas.

Most of the reviews (84%; 11/13) addressed nutrition and hydration; these were often embedded within staff training and environmental interventions, focusing on enteral feeding, mealtime assistance and environmental and behavioural support. Conversely, medical management of symptoms, and diagnosis and prognosis were rarely reported (4%; 3/13), with no interventions specifically targeting pain, pressure ulcers or comorbidities. Interventions with a person-centred component showed positive trends for nutrition and hydration, and activities of daily living, with the caveat that authors consistently recommended the need for the intervention to be adapted to changing preferences as dementia progressed. Interventions with an information and education component also showed positive trends for hydration and nutrition; they also benefitted short-term cognitive outcomes. Whereas interventions focusing on managing neuropsychiatric symptoms were mostly ineffective, except for manual massage. Poor adherence to staff training programmes was linked to time pressures in care settings. Although evidence quality was low and professional roles were often poorly specified, interventions delivered by occupational therapists and physiotherapists demonstrated benefits for balance, mood and slowed deterioration. This reveals a patchy and disjointed evidence base which prioritises a limited range of functional outcomes (favouring nutrition-focused interventions) rather than holistic palliative care needs, with key-guideline relevant symptoms (eg, incontinence, immobility, falls, infections, pain communication) being neglected. Implementation challenges, intervention complexity and outcome standardisation in this population and setting are some of the reasons why evidence was of a low quality and therefore restricted conclusions.

## Discussion

This umbrella review identified 13 reviews which summarised the evidence on physical interventions for people living with advanced dementia, including three meta-analyses. Interventions on the physical body focused on food intake (ie, enteral feeding, supplements, additional food), physical exercise and massages. Interventions on the physical environment were varied and included adaptive equipment and assistance with eating, staff training and altering the environment (eg, adding music). There was very low certainty of the evidence across all included reviews regarding the impact of the interventions on the stated outcomes. Problems such as pressure ulcers and pain management were not identified as outcomes in the meta-synthesis, which was an unexpected finding, despite being key clinical issues that are prevalent in the last months of life for someone living with dementia.^22^ Pressure ulcers were indicated as a risk for enteral feeding.^22^

A key finding from this review is that there are clear gaps in the current evidence base, suggesting that palliative care and dementia care have not yet fully converged. In particular, there is a need for more high-quality, intervention-based research on the medical management of symptoms such as pain, which, despite being well studied in the wider palliative care field for other diseases such as cancer,^36^ remains under-examined in dementia—especially in advanced stages.^37 38^ Moreover, the existing evidence is heavily concentrated in a small number of domains—most notably nutrition and hydration. No eligible systematic reviews were identified for several priority areas that matter greatly to family carers, including bowel and bladder management, which are often triggers for care-home admission^39^; and skin integrity and pressure damage, where risks increase sharply in severe dementia as mobility declines, particularly when individuals become bed-bound. Evidence is also absent regarding approaches that support or promote independence in the later stages of dementia. Together, these gaps highlight the significant need to bring palliative care and dementia care closer together to develop, test and implement effective interventions that address the full spectrum of end-of-life needs for people living with dementia.

Overall, the lack of meta-analyses and the high number of included studies that were considered a high risk of bias prevent any definitive recommendations regarding physical interventions for people with advanced dementia. The evidence was consistent across all reviews that included studies of poor quality and all of them recommended better research methods and study designs as a future priority.

The paucity of high-quality studies is caused by a variety of factors. It is well documented that there is hesitancy when conducting research with such a vulnerable population who lack capacity and are also at the end of life due to ethical, regulatory and consent challenges.^40^ Consequently, this population is often excluded from research, particularly randomised controlled trials, and therefore studies often have smaller samples and weaker study designs.^41^ The complexity of the care environment makes intervention standardisation challenging and workforce-related barriers often affect fidelity.^42^

For reviews that report a meta-analysis, only one review consistently included more than one study in the analysis. The NHS Ambitions Framework^43^ and the NHS 10-year plan^10^ call for the same action: better joined up and holistic care, addressing the whole person’s needs. The updated James Lind Alliance in palliative and end of life care^44^ has as their number one priority, the improvement of care for those living with dementia and those who care for them. This umbrella review highlights the lack of high-quality evidence on physical interventions across palliative dementia care domains and suggests that much more work is needed to better understand how the aims outlined across government policy can be met.

Despite this, the review allows several cautious conclusions to be drawn. The strongest and most consistent finding relates to enteral tube feeding. Evidence from two Cochrane reviews indicates no demonstrable benefit of enteral feeding for people with severe dementia, alongside evidence of potential harm, including a clinically significant increased risk of pressure ulcers. Taken together, this supports the conclusion that enteral tube feeding should not be recommended as a physical intervention in severe dementia.

Beyond enteral feeding, evidence for effectiveness remains limited and intervention-specific. There is low-to-moderate evidence that staff training and education programmes may improve mealtime processes, including reducing feeding difficulties and increasing eating time.^29^

Manual massage is the only physical intervention with pooled quantitative evidence suggesting benefit, with meta-analytical findings indicating improvements in agitation and depressive symptoms.

In contrast, evidence for other physical interventions, including hydration strategies and physical exercise programmes, is inconsistent and insufficient to demonstrate effectiveness. While some studies report positive trends, high risk of bias, outcome heterogeneity and limited end-of-life relevance preclude firm conclusions.

Overall, the available evidence provides clearer guidance on interventions that lack benefit or may cause harm than on those that improve physical outcomes at the end of life. Most physical interventions for people with dementia nearing the end of life remain underinvestigated, with key physical symptoms and palliative care priorities largely neglected.

### Strengths and limitations

This umbrella review is the first to combine the evidence from dementia and palliative care literature for physical interventions for people living with dementia. It has included both qualitative and quantitative data in the synthesis and evidence published in all languages. However, using the methodologies of an umbrella review does have limitations. The search terms were not specific for physical interventions, so it is possible that some systematic reviews may not have been identified. There was no evidence from lower- or middle-income countries which limits the global generalisability of these findings.

## Conclusion

This review highlights that physical-care interventions towards the end of life for people living with dementia have predominantly focused on maintaining well-being and functional ability, with particular emphasis on nutrition and hydration. Despite the low certainty of the evidence, some cautious conclusions can be drawn. Enteral tube feeding shows no benefit and potential harm in severe dementia, while staff training for mealtime care and manual massage demonstrates limited evidence of benefit, with most other physical interventions remaining inconclusive. However, notable gaps remain in the evidence base, particularly in relation to holistic assessment, carer support and the medical management of symptoms. Existing studies are also heavily concentrated in residential care settings, with far less attention paid to acute and community contexts. Addressing these gaps is essential to ensure that people living with dementia are supported, either directly or through adaptations to their environment, to live well until they die.

## Supplementary material

10.1136/spcare-2025-006058 online supplemental file 1

10.1136/spcare-2025-006058 online supplemental file 2

10.1136/spcare-2025-006058 online supplemental file 3

10.1136/spcare-2025-006058 online supplemental file 4

## Data Availability

All data relevant to the study are included in the article or uploaded as supplementary information.
