# Supplementary material for: What is the evidence for physical interventions for people living with advanced dementia in the community? A meta-review
Source: BMJ Support Palliat Care. 2026 Feb 16;16(5):e006058. doi: 10.1136/spcare-2025-006058 (PMC13539838; doi:10.1136/spcare-2025-006058)
Supplement: Supplementary file 1 [file spcare-16-5-s001.docx]

**Supplementary material 1: Search strategy for medline**

| Database | Section | MeSH Term (Medline) | Syntax |
| --- | --- | --- | --- |
| Medline | 1. Population | Dementia/ | dementia/ or alzheimer disease/ or cognition disorders/ or (dement* or alzheim* or "cognitive disorder*").tw. |
|  |  | Alzheimer disease/ |  |
|  |  | Cognition disorders/ |  |
|  | 2a. Care Provision 1: Palliative | Hospice care/ | hospice care/ or palliative care/ or terminal care/ or hospice and palliative care nursing/ or advance care planning/ or hospice.mp. and palliative care nursing/ or ("end of life*" or "hospice*" or "advance care plan*" or "palliat*").tw. |
|  |  | Palliative care/ |  |
|  |  | Terminal care/ |  |
|  |  | Hospice and Palliative care nursing/ |  |
|  |  | Advance care planning/ |  |
|  | 2b. Care Provision 2: Care at home | Home health nursing/ | home health nursing/ or home health aides/ or case management/ or homebound persons/ or home care services/ or social support/ or ("support services").tw |
|  |  | Home health aides/ |  |
|  |  | Case management/ |  |
|  |  | Homebound persons/ |  |
|  |  | Home care services/ |  |
|  |  | Social support/ |  |
|  | 2c. Care Provision 3: Institutional | Long term care/ | Long term care/ or nursing home/ or homes for the aged/ or ("care home* or "nursing home" or "long term care").tw |
|  |  | Nursing home/ |  |
|  |  | Homes for the aged/ |  |
|  | 2d. Care Provision 4: Caregivers | Caregivers/ | caregivers/ or ("caregiver").tw |
|  | 3. Study Design | Review/ | review/ or meta-analysis/ or systematic review/ or (Review or meta-analysis or research report).sh. or (overview$ or review or synthesis or summary or cochrane or analysis).ti. or (meta-review or metareview).ti. or (meta-review or metareview).ab. or ((overview$ or reviews) and (systematic or cochrane)).ti. or (reviews adj2 (meta or published or included or summar$)).ab. or (cochrane review$ ab or (evidence and (reviews or metaanalyses))).ti. or umbrella review.ab. or (reviews or meta-analyses or articles or umbrella).ti. |
|  |  | Meta-analysis/ |  |
|  |  | Systematic review/ |  |
