# Supplementary material for: What is the evidence for physical interventions for people living with advanced dementia in the community? A meta-review
Source: BMJ Support Palliat Care. 2026 Feb 16;16(5):e006058. doi: 10.1136/spcare-2025-006058 (PMC13539838; doi:10.1136/spcare-2025-006058)
Supplement: Supplementary file 2 [file spcare-16-5-s002.docx]

**Supplementary material 2 GRADE interpretation**

GRADE evidence assessment

| **Study outcomes** | | | | **Certainty of Evidence** | | | | | | | | |
| --- | --- | --- | --- | --- | --- | --- | --- | --- | --- | --- | --- | --- |
| **Outcome** | **studies** | **Number of outcomes** | **Number of interventions** | **Risk of bias** | **Inconsistency** | **indirectness** | **Imprecision** | **Publication bias** | **Large effect** | **Plausible confounding** | **Dose response gradient** | **Overall certainty** |
| Agitation | 6 | 1 | 1 | Serious | Serious | Serious | Very serious | undetected | no | would reduce demonstrated effect | no | very low |
| Behaviour | 4 | 4 | 3 | Very Serious | Serious | Very serious | Extremely serious | undetected | no | would suggest spurious effect | no | very low |
| Cognition | 2 | 2 | 2 | Not serious | Very serious | Very serious | Extremely serious | undetected | no | would suggest spurious effect | no | very low |
| Dementia | 1 | 1 | 1 | Very Serious | Very serious | Not serious | Not serious | undetected | no | would suggest spurious effect | no | very low |
| Depression | 5 | 3 | 2 | Very Serious | Very serious | Serious | Very serious | undetected | no | would suggest spurious effect | no | very low |
| Falls | 1 | 1 | 1 | Very Serious | Very serious | Not serious | Not serious | undetected | no | would suggest spurious effect | no | very low |
| Food intake | 14 | 11 | 7 | Not serious | Very serious | Very serious | Extremely serious | undetected | no | would suggest spurious effect | no | very low |
| Function | 5 | 4 | 2 | Very Serious | Serious | Not serious | Extremely serious | undetected | no | would suggest spurious effect | no | very low |
| Muscle mass | 3 | 3 | 1 | Not serious | Very serious | Not serious | Very serious | undetected | no | would suggest spurious effect | no | very low |
| Nutrition | 2 | 2 | 2 | Not serious | Very serious | Very serious | Serious | undetected | no | would suggest spurious effect | no | very low |
| QoL | 1 | 1 | 1 | Not serious | Very serious | Not serious | Very serious | undetected | no | would suggest spurious effect | no | very low |
| Weight | 11 | 8 | 4 | Not serious | Very serious | Very serious | Extremely serious | undetected | no | would suggest spurious effect | no | very low |

**Interpretation of the GRADE**

| **Domain** | **Options** | **Interpretation** | **Further detail** |
| --- | --- | --- | --- |
| Risk of bias | Not serious | Low | by the quality rating |
|  | Serious | Moderate |  |
|  | Very serious | High |  |
| Inconsistency | Not serious | Similar point estimates; overlap with Cis; stat test for heterogeneity not significant; I2 small |  |
|  | Serious | 2 points above not met |  |
|  | Very serious | 3 or more points not met |  |
| indirectness | Not serious | Same intervention; same population; same outcome; same comparator group | |
|  | Serious | 2 points above not met |  |
|  | Very serious | 3 or more points not met |  |
| Imprecision | Not serious | Ci is not wide nor cross the threshold of interest | <https://www.sciencedirect.com/science/article/pii/S0895435622001871#:~:text=Using%20the%20CI%20approach%2C%20when,the%20two%20boundaries%20of%20CI> |
|  | Serious | CI is either wide or crosses the threshold of interest |  |
|  | Very serious | CI is wider and considerably cross the thresholds of interest |  |
|  | Extremely serious | CI is very wide and the two boundaries of CI suggest very different inferences | |
| Publication bias | undetected | Addressed in the review and not identified |  |
|  | strongly suspected | Either not reported or identified. |  |
| Large effect | yes | RR >2 or <0.5; very large RR >5, <0.2 | <https://gdt.gradepro.org/app/handbook/handbook.html#h.6wuuudis64a8> |
|  | no |  |  |
| Plausible confounding | no |  |  |
|  | would reduce demonstrated effect | |  |
|  | would suggest spurious effect | |  |
| Dose response gradient | yes |  |  |
|  | no |  |  |
