# Supplementary material for: What is the evidence for physical interventions for people living with advanced dementia in the community? A meta-review
Source: BMJ Support Palliat Care. 2026 Feb 16;16(5):e006058. doi: 10.1136/spcare-2025-006058 (PMC13539838; doi:10.1136/spcare-2025-006058)
Supplement: Supplementary file 3 [file spcare-16-5-s003.docx]

Supplementary material 3: AMSTAR and EMTREQ

| **AMSTAR** | **Research question and inclusion criteria** | **Protocol** | **Study design** | **Search strategy** | **Study selection - duplication** | **Extraction - duplication** | **Excluded studies** | **Included studies** | **Risk of bias** | **Funding sources** | **Meta-analysis – suitable methods** | **Meta-analysis – risk of bias** | **Interpretation – risk of bias** | **Discussion of heterogeneity** | **Publication bias** | **Conflict of interest** |
| --- | --- | --- | --- | --- | --- | --- | --- | --- | --- | --- | --- | --- | --- | --- | --- | --- |
| Brett 2016 | Yes | No | No | Yes | No | No | Yes | Partial Yes | Yes | No | Meta-analysis not performed | Meta-analysis not performed | Yes | Yes | No Meta-analyses conducted | Yes |
| Bunn 2015 | Yes | Partial Yes | No | Yes | Yes | Yes | Yes | Yes | Yes | No | Meta-analysis not performed | Meta-analysis not performed | Yes | Yes | No Meta-analyses conducted | Yes |
| Forbes 2008 | Yes | Yes | Yes | Yes | Yes | Yes | Yes | Partial Yes | Yes | No | Yes | Yes | Yes | Yes | No | Yes |
| Herke 2018 | Yes | Yes | Yes | Yes | Yes | Yes | Yes | Yes | Yes | Yes | Yes | Yes | Yes | Yes | Yes | Yes |
| Konno 2014 | Yes | Partial Yes | Yes | Partial Yes | Yes | Yes | Partial Yes | No | Partial Yes | No | Meta-analysis not performed | Meta-analysis not performed | Yes | Yes | No Meta-analyses conducted | Yes |
| Liu 2014 | Yes | Partial Yes | Yes | Partial Yes | Yes | Yes | Yes | Yes | Yes | No | Meta-analysis not performed | Meta-analysis not performed | Yes | No | No Meta-analyses conducted | Yes |
| Lui 2015 | Yes | Partial Yes | Yes | Yes | Yes | No | Yes | Yes | No | No | Meta-analysis not performed | Meta-analysis not performed | Yes | No | No Meta-analyses conducted | No |
| Margenfeld 2019 | Yes | Partial Yes | Yes | Yes | No | No | Partial Yes | Partial Yes | Yes | No | Yes | Yes | Yes | No | Yes | Yes |
| Sampson 2009 | Yes | Yes | Yes | Yes | No | No | Yes | Yes | Yes | No | Meta-analysis not performed | Meta-analysis not performed | Yes | Yes | No Meta-analyses conducted | Yes |
| Barret et al. 2021 | Yes | Yes | Partial yes | Partial yes | Yes | Yes | No | Yes | Yes | No | Meta-analysis was not performed | Meta-analysis was not performed | Yes | Partial yes | No Meta-analyses performed | Yes (no conflict of interest) |
| Davies et al. 2021 | Yes | Yes | Partial yes | Yes | Yes | Yes | Yes | Yes | Yes | Yes | Meta-analysis was not performed | Meta-analysis was not performed | Yes | Yes | No Meta-analyses performed | Yes (no conflict of interest) |

| **ENTREQ** | **Aim** | **Plan for analysis** | **Search strategy** | **Inclusion criteria** | **Data sources** | **Literature search** | **Two independent screeners** | **Study Characteristics** | **Flow diagram** | **Number of studies included** | **Rationale for included studies** | **Quality appraisal** | **Appraisal - independent** | **Results of quality appraisal** | **Extraction** | **Software** | **Two reviewers coding** | **Appropriate methods?** | **Comparisons** | **Process of identifying themes** | **Synthesis output** |
| --- | --- | --- | --- | --- | --- | --- | --- | --- | --- | --- | --- | --- | --- | --- | --- | --- | --- | --- | --- | --- | --- |
| Barrado-Martin 2021 | Yes | Yes | Yes | Yes | Yes | Yes | Yes | Yes | Yes | Yes | Yes | Yes | Yes | Yes | Yes | No | Yes | Yes | The team met to discuss and explore relationships and hierarchies between codes, devising themes across the data. Preliminary themes were shared and discussed with all authors for further discussion and refinement. | Inductive | Yes |
| Wilson 2020 | Yes | No | Yes | Yes | Yes | No | No | Yes | No | No | No | No | No | No | No | No | No | No | Doesn’t specify | Inductive | Yes |
