# Supplementary material for: What is the evidence for physical interventions for people living with advanced dementia in the community? A meta-review
Source: BMJ Support Palliat Care. 2026 Feb 16;16(5):e006058. doi: 10.1136/spcare-2025-006058 (PMC13539838; doi:10.1136/spcare-2025-006058)
Supplement: Supplementary file 4 [file spcare-16-5-s004.docx]

**Supplementary material 4: Graphical Representation of Overlap for OVErviews (GROOVE)**

|  | | Sampson |  |  |  |  |  |  |  |  |  |  |  |  |  |  |  |  |  |  |  |  |  |  |  |  |  |  |  |  |  |
| --- | --- | --- | --- | --- | --- | --- | --- | --- | --- | --- | --- | --- | --- | --- | --- | --- | --- | --- | --- | --- | --- | --- | --- | --- | --- | --- | --- | --- | --- | --- | --- |
|  | |  | Davies | Barrado-Martin | Bunn | Wilson | Liu 2014 | Liu 2015 | Herke | Brett |  |  |  |  |  |  |  |  |  |  |  |  |  |  |  |  |  |  |  |  |  |
|  | |  |  |  |  |  |  |  |  |  | **78** | **=** | **Total nodes (pairs of reviews)** | | | | |  |  |  |  |  |  |  |  |  |  |  |  |  |  |
|  | Davies | 16.7% |  |  |  |  |  |  |  |  | 71 | = | Slight overlap (<5%) | | | | |  |  |  |  |  |  |  |  |  |  |  |  |  |  |
|  | Barrado-Martin | 0.0% | 0.0% |  |  |  |  |  |  |  | 3 | = | Moderate overlap (5% to <10%) | | | | |  |  |  |  |  |  |  |  |  |  |  |  |  |  |
|  | Bunn | 0.0% | 0.0% | 0.0% |  |  |  |  |  |  | 2 | = | High overlap (10% to <15%) | | | | |  |  |  |  |  |  |  |  |  |  |  |  |  |  |
|  | Wilson | 0.0% | 0.0% | 0.0% | 6.1% |  |  |  |  |  | 2 | = | Very High overlap (≥15%) | | | | |  |  |  |  |  |  |  |  |  |  |  |  |  |  |
|  | Liu 2014 | 0.0% | 0.0% | 0.0% | 2.3% | 3.0% |  |  |  |  | Forbes | Barrett | Margenfeld |  |  |  |  |  |  |  |  |  |  |  |  |  |  |  |  |  |  |
|  | Liu 2015 | 0.0% | 0.0% | 0.0% | 0.0% | 0.0% | 13.3% |  |  |  |  |  |  |  |  |  |  |  |  |  |  |  |  |  |  |  |  |  |  |  |  |
|  | Herke | 0.0% | 0.0% | 0.0% | 0.0% | 0.0% | 3.3% | 10.5% |  |  |  |  |  |  |  |  |  |  |  |  |  |  |  |  |  |  |  |  |  |  |  |
|  | Brett | 0.0% | 0.0% | 0.0% | 0.0% | 0.0% | 0.0% | 0.0% | 0.0% |  |  |  |  |  |  |  |  |  |  |  |  |  |  |  |  |  |  |  |  |  |  |
|  | Forbes | 0.0% | 0.0% | 0.0% | 0.0% | 0.0% | 0.0% | 0.0% | 0.0% | 5.9% |  |  |  |  |  |  |  |  |  |  |  |  |  |  |  |  |  |  |  |  |  |
|  | Barrett | 0.0% | 0.0% | 0.0% | 0.0% | 0.0% | 0.0% | 0.0% | 0.0% | 23.3% | 0.0% |  |  |  |  |  |  |  |  |  |  |  |  |  |  |  |  |  |  |  |  |
|  | Margenfeld | 0.0% | 0.0% | 0.0% | 0.0% | 0.0% | 0.0% | 0.0% | 0.0% | 0.0% | 0.0% | 0.0% |  |  |  |  |  |  |  |  |  |  |  |  |  |  |  |  |  |  |  |
|  | Konno | 0.0% | 0.0% | 0.0% | 0.0% | 0.0% | 5.1% | 0.0% | 0.0% | 0.0% | 0.0% | 0.0% | 0.0% |  |  |  |  |  |  |  |  |  |  |  |  |  |  |  |  |  |  |
|  |  |  |  |  |  |  |  |  |  |  |  |  |  |  |  |  |  |  |  |  |  |  |  |  |  |  |  |  |  |  |  |
|  |  |  |  |  |  |  |  |  |  |  |  |  |  |  |  |  |  |  |  |  |  |  |  |  |  |  |  |  |  |  |  |
|  |  |  |  |  |  |  |  |  |  |  |  |  |  |  |  |  |  |  |  |  |  |  |  |  |  |  |  |  |  |  |  |
